# Machine Learning and Abdominal Aortic Aneurysm: A New Paradigm in Prediction and Prognosis after Endovascular Aneurysm Repair

**DOI:** 10.3400/avd.ra.25-00120

**Published:** 2026-01-20

**Authors:** Toshiya Nishibe, Tsuyoshi Iwasa, Shoji Fukuda, Tomohiro Nakajima, Shinichiro Shimura, Masayasu Nishibe, Alan Dardik

**Affiliations:** 1 Department of Medical Informatics and Management, Hokkaido Information University, Ebetsu, Hokkaido, Japan; 2 Department of Cardiovascular Surgery, Tokyo Medical University, Tokyo, Japan; 3 Department of Cardiovascular Surgery, Sapporo Medical University, Sapporo, Hokkaido, Japan; 4 Department of Cardiovascular Surgery, Toho University Ohashi Medical Center, Tokyo, Japan; 5 Department of Surgery, Eniwa Midorino Clinic, Eniwa, Hokkaido, Japan; 6 Department of Vascular Surgery, Icahn School of Medicine at Mount Sinai, New York, USA

**Keywords:** artificial intelligence, machine learning, abdominal aortic aneurysm, prognosis, endovascular aneurysm repair

## Abstract

Artificial intelligence (AI) and machine learning (ML) are transforming vascular surgery by enabling precise risk stratification, individualized treatment planning, and improved prognostic prediction. In abdominal aortic aneurysm (AAA) management, ML algorithms integrate complex clinical and imaging data to estimate survival, guide procedural decisions, and identify key factors influencing aneurysm remodeling. These models outperform traditional statistical approaches by capturing nonlinear interactions among variables such as nutritional status, immune function, and anatomical features. Despite these advances, challenges remain. Many studies rely on single-center datasets, raising concerns about overfitting and limited generalizability. The use of black-box models can hinder clinical trust due to limited interpretability. However, recent developments in multicenter data collection and explainable AI techniques are improving model robustness and transparency. As these tools continue to evolve, ML is poised to contribute meaningfully to precision vascular care. By supporting more individualized and data-informed decision-making, ML has the potential to enhance long-term outcomes and guide the future of AAA management after endovascular aneurysm repair.

## 1. Introduction

**Fig. 1:**
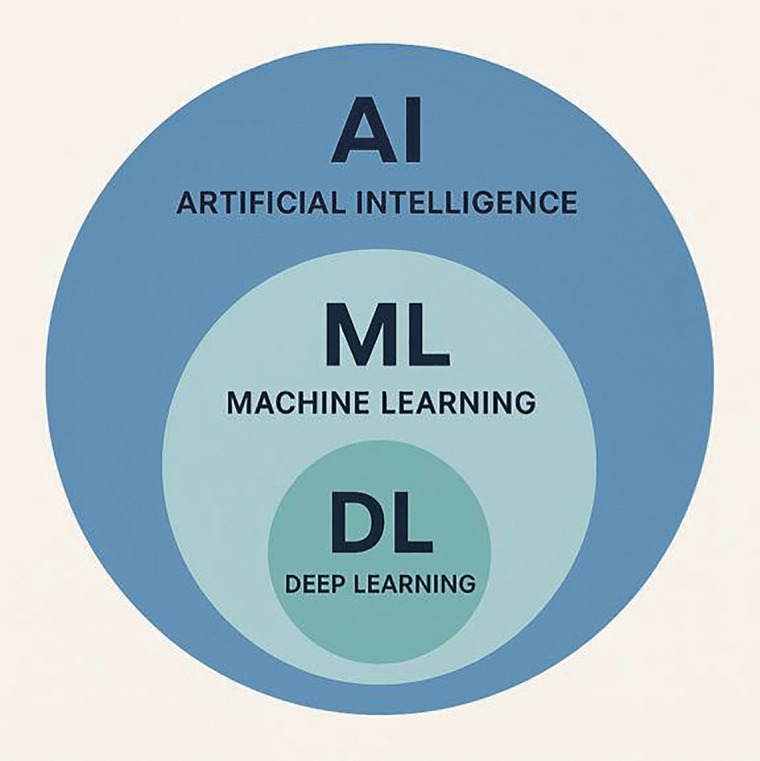
Hierarchical relationship between AI, ML, and DL. AI refers to systems that emulate human intelligence; ML, a subset of AI, supports data-driven learning and prediction; DL, a subset of ML, uses neural networks to model complex patterns in large datasets. AI: artificial intelligence; ML: machine learning; DL: deep learning

Artificial intelligence (AI), machine learning (ML), and deep learning (DL) are increasingly transforming clinical medicine.^1)^ AI comprises ML, with DL constituting a potent subset that utilizes neural networks for extracting knowledge from large volumes of data (**Fig. 1**). These technologies support automated image interpretation, early disease detection, prognosis prediction, and individualized treatment planning across many clinical specialties.

Vascular surgery includes complex conditions such as aortic aneurysm, peripheral arterial disease, and venous disorders. Patients are often elderly with malnutrition, impaired immune function, and multiple comorbidities, all of which significantly affect postoperative outcomes.^2)^ Traditional statistical models struggle to capture the nonlinear and multidimensional interactions present in these clinical settings.

**Fig. 2:**
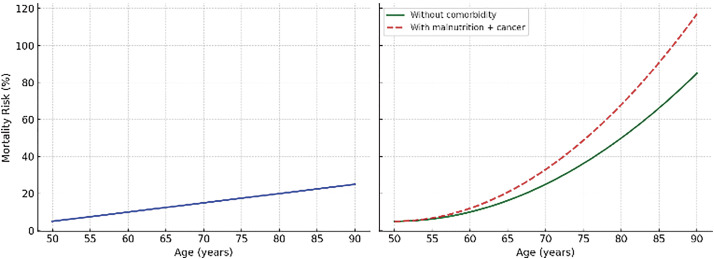
Comparison of linear and nonlinear mortality risk models. (Left) A linear model (traditional statistical models) shows a steady rise in mortality risk with age, assuming independent effects of each variable. (Right) A nonlinear model (machine learning models) demonstrates a steep rise in risk when advanced age, malnutrition, and active cancer coexist, capturing synergistic interactions beyond additive effects.

Linearity assumes that each risk factor affects outcomes independently and proportionally, like age-related mortality risk. Clinical outcomes are rarely so straightforward, where a combination of factors, such as advanced age, malnutrition, and active cancer, can sharply amplify risk beyond the sum of their individual effects, a phenomenon known as nonlinearity (**Fig. 2**).^3,4)^ Such complexity is not well captured by conventional statistical models.

ML algorithms can capture such nonlinear and multidimensional interactions, enabling more accurate prediction of patient outcomes than conventional statistical models.^5)^ By integrating clinical and imaging data, ML supports risk stratification, procedural decision-making, and prognosis estimation.^6)^ These capabilities are particularly valuable in abdominal aortic aneurysm (AAA) management, where patient heterogeneity and anatomical complexity challenge conventional statistical models. This review highlights the emerging role of ML in AAA prognosis, especially after endovascular aneurysm repair (EVAR), and discusses current applications, limitations, and future directions.

## 2. ML Models

**Fig. 3:**
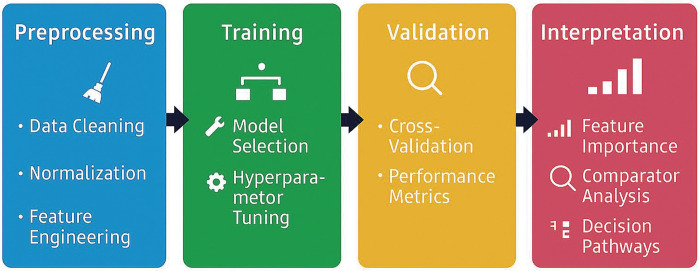
Schematic overview of machine learning pipeline used in abdominal aortic aneurysm research. The workflow comprises 4 key stages: data preprocessing (including cleaning, normalization, and feature engineering), model training with internal cross-validation and hyperparameter tuning, validation using external cohorts and comparator analysis, and interpretation through feature importance metrics and decision pathways.

**Fig. 4:**
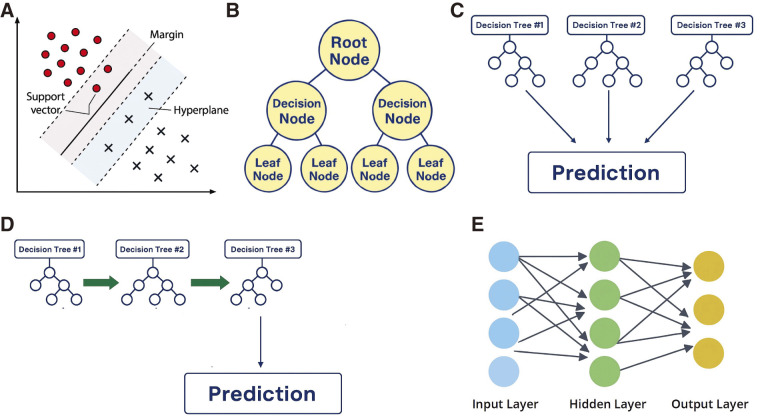
Representative machine learning algorithms for modeling complex data structures. (**A**) Support vector machine: Defines optimal classification boundaries with maximum margin. (**B**) Decision tree: Splits data hierarchically based on feature thresholds. (**C**) Random forest: Aggregates multiple decision trees to enhance robustness and reduce overfitting. (**D**) Gradient boosting: Sequentially improves predictions by focusing on residual errors. (**E**) Neural network: Learns nonlinear relationships through layered node connections.

ML is a core component of AI that enables systems to learn patterns from data without explicit programming. ML is typically implemented using platforms such as Python and libraries like scikit-learn, which support data preprocessing, model development, and performance evaluation (**Fig. 3**).^7)^ ML algorithms are generally classified into 2 main types: supervised learning, which relies on labeled data to predict specific outcomes, and unsupervised learning, which uncovers hidden patterns within unlabeled datasets without predefined categories.^8)^ A wide variety of ML algorithms are available, each suited to specific predictive or classification tasks (**Fig. 4**).^9)^ A decision tree (DT) is a rule-based model that recursively splits data into branches based on feature thresholds. Random forest (RF) and gradient boosting are ensemble ML models that combine multiple DTs; RF aggregates DTs in parallel to reduce variance and improve robustness, while gradient boosting builds DTs sequentially to correct errors and enhance predictive accuracy. Support vector machines (SVMs) classify data by delineating optimal boundaries between categories. Advanced techniques such as artificial neural networks (ANNs) can detect subtle patterns in high-dimensional data that often escape conventional statistical models.

To enhance interpretability and reduce the “black-box” nature, feature importance analysis is often used to quantify the contribution of each variable to model predictions. SHAP (Shapley Additive exPlanations) and LIME (Local Interpretable Model-agnostic Explanations) are increasingly used to explain individual predictions, fostering clinical trust in ML outputs.^10)^

Through these processes, ML models identify key determinants, enable patient stratification, and enhance outcome prediction with greater accuracy than conventional statistical models.^11)^

## 3. Trends in ML for Vascular Surgery

The use of ML in vascular surgery has expanded rapidly over the past 2 decades. A systematic review by Li et al. identified 212 original studies published between January 1991 and March 2021, with a sharp increase in volume after 2016.^12)^ These studies primarily focused on diagnostic support (39%), imaging analysis (35%), and prognostic modeling (26%) across a wide range of vascular conditions, including carotid stenosis, aortic aneurysm and dissection, peripheral artery disease, venous disorders, and others.

Although most studies demonstrated strong predictive performance with a median area under the curve (AUC) value of 0.88 and approximately 80% achieving AUC values above 0.80, the majority were retrospective and single center in design. This raises concerns about bias, overfitting, and limited generalizability. Common limitations included the lack of external validation, incomplete reporting of model development and evaluation processes, and small sample sizes that compromise the robustness of model performance.

These trends are highly relevant to the management of AAA, where patient variability and anatomical complexity limit conventional statistical models. ML provides an alternative by modeling nonlinear patterns and integrating diverse data. The following section summarizes ML applications in predicting AAA prognosis after EVAR.

## 4. ML-Based Prognostic Modeling in AAA and EVAR Management

**Tables 1A–1D** presents an overview of ML-based prognostic frameworks used for risk evaluation in AAA and EVAR.

**Table 1:** Machine learning-based prognostic models in abdminal aortic aneurysm and endovascular aneurysm repair (EVAR) management

A. Aneurysm growth and preoperative risk stratification							
Author (year)	Design	N	Intervention	ML method (s)	Outcome	Performance	Validation
Lee (2018)^13)^	Prospective	94	None	SVM	Sac growth at 12 and 24 months	N/A	k-fold^1^
Lindquist Liljeqvist (2021)^14)^	Retrospective, single center	189	None	RR, LASSO, KNN, SVM, RF, XGBoost, ANN	Sac growth within 4 years	AUC 0.8465^2^	10-fold^1^
Kontopodis (2023)^15)^	Retrospective, single center	40	None	SVM, XGBoost	Sac growth within 36 months	AUC 0.812^2^	7:3 hold-out^3^
Chung (2024)^16)^	Retrospective, database	381	None	XGBoost	Sac outcome (stable/repair-needed/rupture-prone)	AUC 0.90/0.80/0.91^2^	8:2 hold-out^3^
B. Mortality and MACE after EVAR							
Author (year)	Design	N	Intervention	ML method (s)	Outcome	Performance	Validation
Li (2024)^19)^	Retrospective, database	16282	EVAR	XGBoost, RF, SVM, NB, ANN, LR	30-day MACE	AUC 0.95^2^, Acc 88%^2^	7:3 hold-out^3^
Li (2023)^20)^	Retrospective, database	63655	EVAR	XGBoost, RF, SVM, NB, ANN, LR	1-year mortality	AUC 0.96^2^, Acc 90%^2^	7:3 hold-out^3^
Thompson (2025)^21)^	Retrospective, single center	925	OSR/EVAR	RF	2-year survival	AUC 0.88^2^, Acc 92.6%^2^	10-fold^1^
Nishibe (2025)^22)^	Retrospective, single center	169	EVAR	DT	3-year mortality	Acc 68.7%^2^	5-fold^1^
Nishibe (2025)^23)^	Retrospective, single center	142	EVAR	DT	5-year survival	AUC 0.82^2^, Acc 76.1%^2^	5-fold^1^
Nishibe (2025)^24)^	Retrospective, single center	169	EVAR	RF	3-year mortality	AUC 0.76^2^, Acc 73.9%^2^	5-fold^1^
C. Postoperative complications and reintervention after EVAR							
Author (year)	Design	N	Intervention	ML method (s)	Outcome	Performance	Validation
Karthikesalingam (2015)^27)^	Retrospective, multicenter	761	EVAR	ANN	5-year endograft complication/mortality	C-statistic 0.776/0.699^2^	External^4^
Attallah (2014)^28)^	Retrospective, multicenter	146	EVAR	ANN	Reintervention	AUC 0.702^2^	External^4^
Attallah (2017)^29)^	Retrospective, multicenter	743	EVAR	ANN	Reintervention	AUC 0.666^2^	External^4^
Kordzadeh (2021)^30)^	Retrospective, single center	250	EVAR	ANN	Endoleaks (Type I–III)	Acc 96.3%/0.3%/92.6%^2^	1:1 hold-out^3^
D. Sac shrinkage after EVAR							
Author (year)	Design	N	Intervention	ML method (s)	Outcome	Performance	Validation
Nishibe (2025)^32)^	Retrospective, single center	119	EVAR	DT	Sac shrinkage at 2 years	Acc 60.4%^2^	5-fold^1^
Huang (2025)^33)^	Retrospective, single center	164	EVAR	SVM	Sac shrinkage	AUC 0.87^2^	7:3 hold-out^3^

^1^k-fold cross-validation: Dataset is split into k subsets; each subset is used once as a test set while the remaining k–1 subsets form the training set.

^2^Performance metrics: AUC and Acc are used to evaluate model discrimination and prediction correctness.

^3^Hold-out validation: Dataset is split into fixed training and testing sets, commonly in ratios such as 7:3 or 8:2.

^4^External validation: Model tested on independent datasets from different institutions or populations.

SVM: support vector machine; RR: ridge regression; LASSO: least absolute shrinkage and selection operator; KNN: k-nearest neighbor; RF: random forest; XGBoost: extreme gradient boost; ANN: artificial neural network; NB: Naive Bayes; LR: logistic regression; DT: decision tree; OSR: open surgical repair; MACE: major adverse cardiovascular events; AUC: area under the curve; Acc: accuracy

### Aneurysm growth and preoperative risk stratification (Table 1A)

ML has significantly advanced the prediction of aneurysm growth or rupture and the stratification of preoperative risk in patients with AAA. By integrating clinical, morphological, and biomechanical data, ML models can identify subtle patterns that are often overlooked by conventional statistical models.

Several studies have demonstrated the utility of ML in this domain. Lee et al. applied kernel-based support vector regression to predict aneurysm growth from baseline flow-mediated dilatation, achieving individualized predictors of expansion rates.^13)^ Similarly, Lindquist Liljeqvist et al. combined semi-automated diameter measurements with geometric and biomechanical modeling, showing that ML-assisted regression and stratification algorithms could improve the prediction of aneurysm growth, surgical indication, and rupture risk in small AAAs.^14)^

Kontopodis et al. developed a hybrid model of extreme gradient boost (XGBoost) and SVM to classify AAAs into high- and low-growth groups.^15)^ This approach integrated a wide range of features, including clinical, biological, morphologic, and biomechanical data, highlighting the strength of ML in managing multidimensional datasets. Chung et al. introduced an ML-based classifier that integrated multivariable patient data to categorize AAAs as stable, repair-needed, or rupture-prone.^16)^ This model offered actionable insights for clinicians, enabling more targeted surveillance and timely interventions.

These studies highlight the clinical utility of ML in enhancing preoperative risk stratification by integrating multimodal data and identifying patients at increased risk of aneurysm progression or rupture, thereby supporting individualized management and timely intervention.

### Mortality and major adverse cardiovascular events after EVAR (Table 1B)

EVAR has become a widely accepted alternative to open surgical repair (OSR), providing reduced perioperative mortality. However, long-term survival remains suboptimal, while early postoperative mortality and major adverse cardiovascular events (MACE) continue to present significant challenges.^17,18)^ Recently, ML has emerged as a powerful tool for predicting these outcomes and supporting perioperative risk management.

Li et al. developed ML models using 36 preoperative variables to predict 30-day MACE after EVAR. Among the algorithms tested, XGBoost demonstrated superior accuracy and consistent performance across diverse patient subgroups.^19)^ Subsequently, the same group used XGBoost to predict 1-year mortality, identifying key predictors such as poor functional status, preoperative dialysis, and unfitness for OSR.^20)^ These models outperformed traditional logistic regression and offered actionable insights for clinical decision-making.

Thompson et al. used an RF model to predict 2-year survival following elective AAA repair, highlighting physiological variables such as anaerobic threshold and preoperative hemoglobin as significant predictors.^21)^ These findings underscore the value of ML in capturing complex interactions among physiological and demographic factors.

**Fig. 5:**
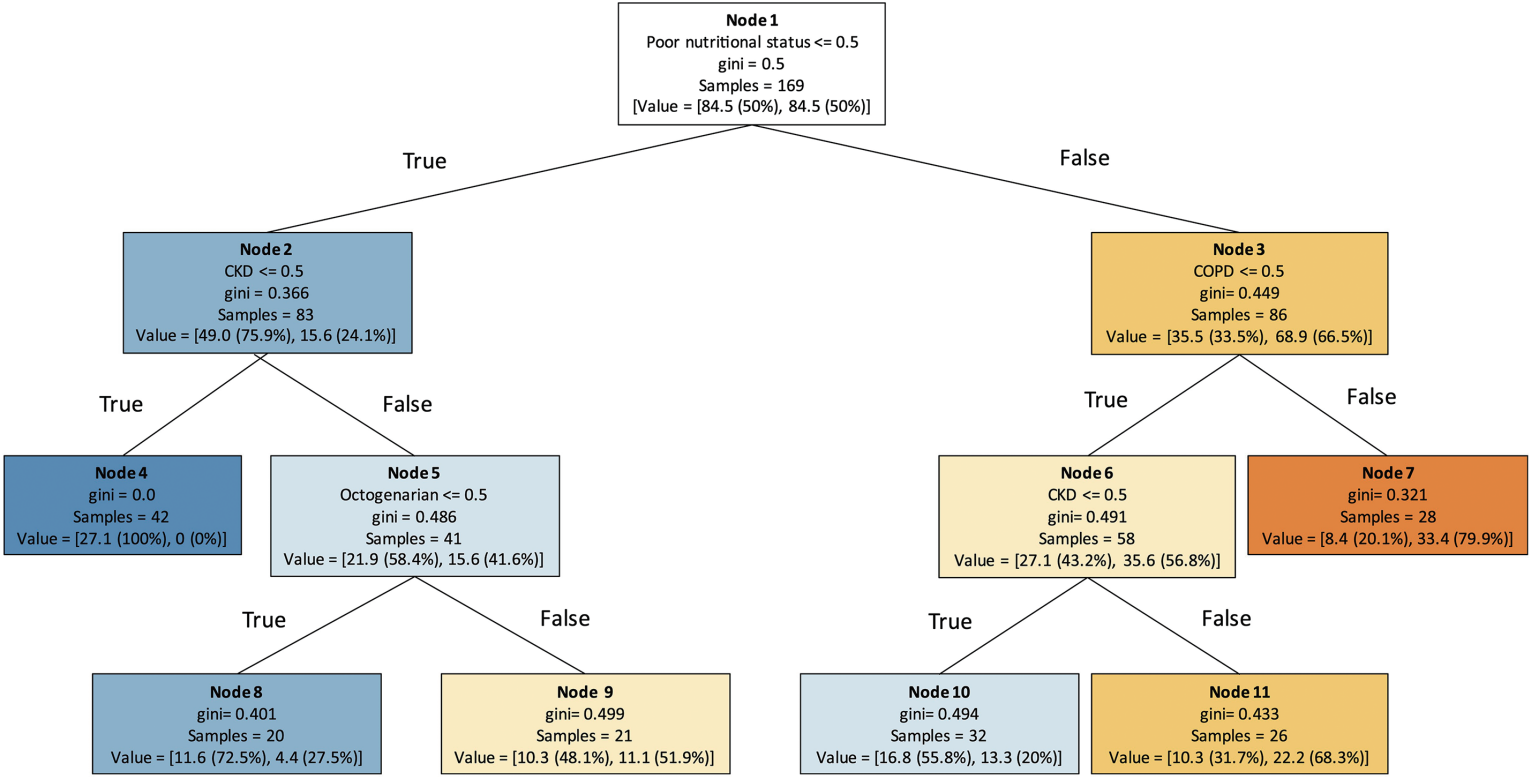
A decision tree model for predicting 3-year mortality following endovascular aneurysm repair. This decision tree, generated using the classification and regression trees method, visualizes the hierarchical relationship between 3-year mortality and 4 variables identified in univariable analysis: octogenarian, poor nutritional status, COPD, and CKD. Blue nodes represent a higher probability of survival, while orange nodes indicate increased mortality risk. Reproduced from Nishibe et al.^22)^ with permission from Elsevier. © 2025 Elsevier Ltd. All rights reserved. COPD: chronic obstructive pulmonary disease; CKD: chronic kidney disease

**Fig. 6:**
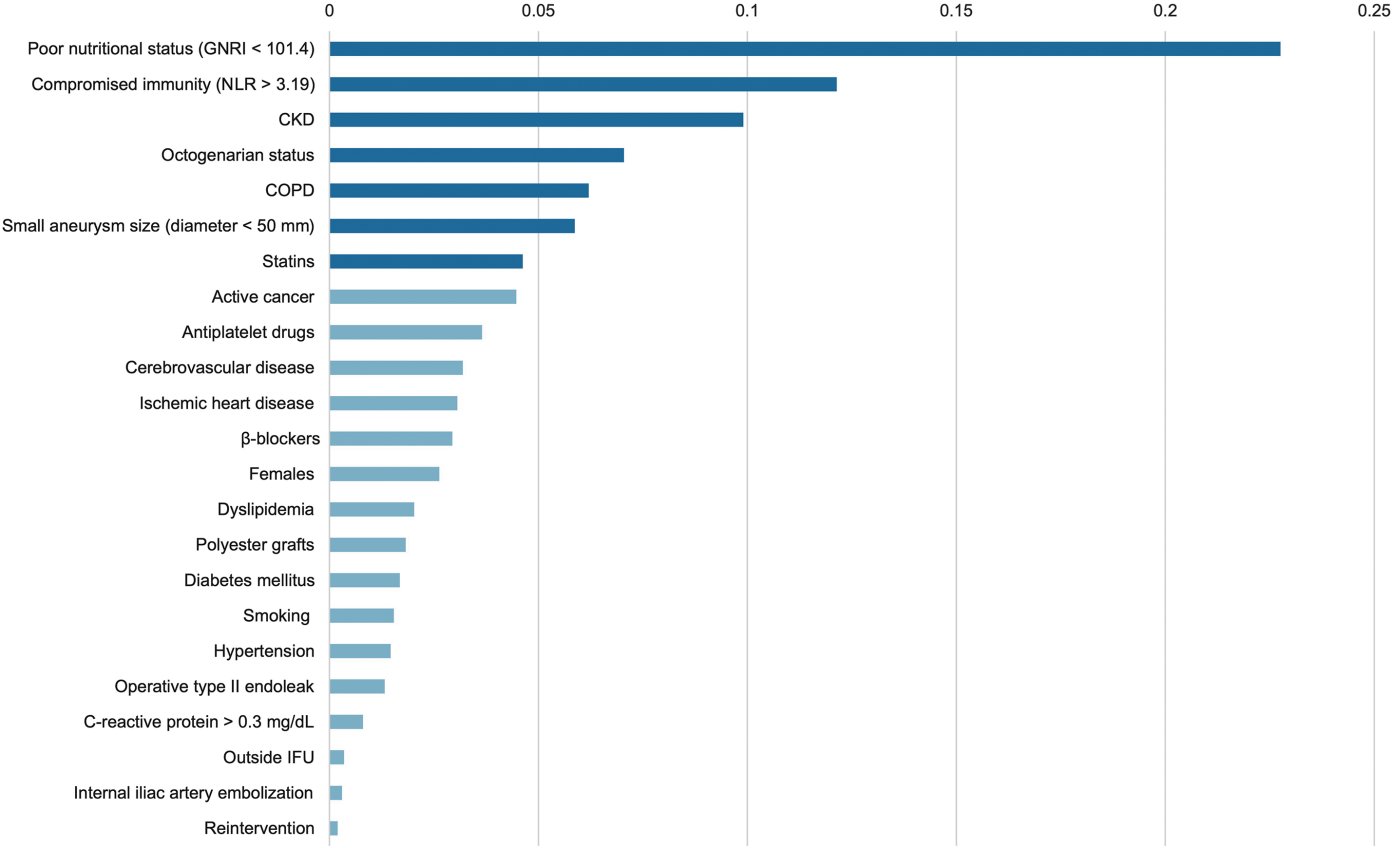
Feature importance analysis of 3-year survival using a random forest model. Seven key predictors are highlighted by dark blue bars, indicating their relative contribution to survival prediction. Variables include GNRI, CKD, COPD, IFU compliance, and NLR. Reproduced from Nishibe et al.^23)^ GNRI: geriatric nutritional risk index; CKD: chronic kidney disease; COPD: chronic obstructive pulmonary disease; IFU: instruction for use; NLR: neutrophil-to-lymphocyte ratio

Our own research further demonstrated the utility of ML in predicting mortality after EVAR. Using a Classification And Regression Tree (CART), one of the DT algorithms, we identified poor nutritional status as the most influential predictor of 3-year mortality, followed by chronic kidney disease (CKD), chronic obstructive pulmonary disease (COPD), and advanced age (**Fig. 5**).^22)^ We also developed an RF model that achieved high predictive accuracy for 3-year survival, with feature importance analysis revealing compromised immunity, small aneurysm size, and statin use as additional key predictors (**Fig. 6**).^23)^ Separately, we extended this approach to predict 5-year survival, again confirming the dominant role of nutritional status, immune function, and comorbidities.^24)^

Taken together, these studies suggest that ML algorithms can provide nuanced, individualized risk assessments that go beyond conventional scoring systems such as Glasgow Aneurysm Score (GAS) and Vascular Governance North West (VSGW);^25,26)^ By their nature, ML models can capture complex interactions among diverse clinical and imaging variables, enabling preoperative identification of high-risk patients and supporting tailored surveillance and clinical decision-making to improve long-term outcomes after EVAR.

### Postoperative stent graft-related complications (Table 1C)

Despite the minimally invasive nature of EVAR, postoperative complications remain a significant concern. These include endoleaks, stent graft migration, and structural failure, all of which can lead to reintervention and increased mortality. ML has shown promise in predicting these adverse outcomes and supporting long-term surveillance strategies.

Karthikesalingam et al. demonstrated that ANNs could accurately stratify patients by their 5-year risk of endograft complications and mortality using preoperative aneurysm morphology.^27)^ Their model provided clinicians with a data-driven framework for anticipating long-term risks. Similarly, Attallah and Ma applied a back-propagation ANN to distinguish between high- and low-risk patients for reintervention after EVAR.^28)^ They further developed a feature selection and survival modeling framework that addressed the high censoring typical of post-EVAR datasets, outperforming traditional Cox proportional hazards models.^29)^

Kordzadeh et al. used ANNs to model 26 preoperative variables and predict a range of complications, including endoleaks, graft occlusion and migration, and mortality.^30)^ Their model achieved high overall accuracy and demonstrated the feasibility of ML-based surveillance planning.

### Sac shrinkage after EVAR (Table 1D)

Aneurysm sac shrinkage is widely regarded as a favorable indicator of successful EVAR and is often used as a surrogate marker of long-term durability.^31)^ Predicting sac shrinkage, however, remains challenging because of the multifactorial nature of aneurysm remodeling. ML has emerged as a valuable tool in this context, capable of uncovering complex, nonlinear relationships among patient characteristics, procedural variables, and postoperative outcomes.

Our group developed a DT-based model to predict sac shrinkage 3 years after EVAR.^32)^ The model identified key predictors such as arterial compliance, baseline aneurysm diameter, and the presence of type II endoleak. These findings highlighted both anatomical and physiological factors influencing sac behavior. Arterial stiffness and type II endoleak status were among the 2 most influential variables, suggesting that ML can capture subtle interactions that may not be apparent through conventional statistical analysis.

In a complementary approach, Huang et al. developed a radiomics-based ML model using preoperative computed tomography angiography data.^33)^ Radiomics refers to the extraction of high-dimensional quantitative features from medical images, such as texture, shape, and intensity patterns. Their model, which incorporated features from the aneurysm sac and surrounding perivascular adipose tissue, achieved high accuracy in predicting sac regression. Notably, the inclusion of perivascular fat characteristics enhanced prognostic performance, underscoring the potential of ML to integrate novel imaging biomarkers into clinical risk models.

These studies demonstrate that ML can provide accurate, individualized predictions of sac shrinkage after EVAR. ML models, leveraging both clinical and imaging data, provide a more nuanced understanding of aneurysm remodeling and may facilitate tailored follow-up strategies, as well as earlier identification of high-risk patients.

## 5. Current Limitations and Future Directions

ML offers considerable promise in predicting outcomes after both EVAR and OSR, although several limitations must be addressed before ML models can be widely adopted in clinical practice. Many existing studies rely on small, single-center datasets, which increase the risk of overfitting and limited generalizability of findings.^34)^ Without external validation, even high-performing models may fail to replicate in broader, more diverse patient populations, although internal validation can help mitigate this risk.^35)^

Another major challenge lies in data quality and completeness. Missing or inconsistent clinical and imaging data can compromise model accuracy and introduce bias. Furthermore, the use of “black-box” algorithms may undermine clinical trust due to their lack of transparency. Clinicians are often reluctant to rely on predictions they cannot interpret or explain to patients.

To overcome these barriers, future research should develop robust, externally validated models trained on large, multicenter datasets that capture diverse patient characteristics, procedural variations, and institutional practices, thereby enhancing reliability and generalizability. Explainable AI techniques, such as SHAP and LIME, can also clarify how individual features influence predictions, supporting transparency and accountability in clinical settings.^10)^

An important future direction is the integration of ML models into real-world clinical workflows. This entails harmonizing data across electronic health record (EHR) systems, ensuring interoperability, and developing user-friendly interfaces that support shared decision-making.^36)^ Ethical and regulatory considerations, including data privacy, algorithmic fairness, and the need for continuous model monitoring and updating, must also be addressed.^37)^

These future directions are summarized in **Table 2**, highlighting key areas such as wearable data integration, hybrid biomechanical–ML models, explainable AI, federated learning, and ethical frameworks.

**Table 2:** Future outlook of machine learning in abdominal aortic aneurysm and EVAR

Research direction	Key focus/expected impact
Wearable and longitudinal data integration	Continuous physiologic and imaging data may enable dynamic aneurysm risk monitoring after EVAR
Hybrid biomechanical–ML models	Combining finite element analysis with ML could enhance the prediction of rupture and sac behavior
Explainable AI	Interpretability tools such as SHAP and LIME improve clinical acceptance and transparency
Federated and multicenter learning	Privacy-preserving collaboration can expand data diversity and model generalizability
Ethical and regulatory frameworks	Addressing bias, governance, and implementation barriers is essential for clinical deployment

ML: machine learning; SHAP: Shapley Additive exPlanations; LIME: Local Interpretable Model-agnostic Explanations; AI: artificial intelligence; EVAR: endovascular aneurysm repair

Finally, incorporating novel data sources, such as wearable sensor data, longitudinal imaging, and biomechanical simulations, may further enhance predictive performance. Hybrid models that integrate ML with domain-specific physiological modeling could provide a more comprehensive understanding of aneurysm sac behavior and treatment response.

ML has demonstrated strong potential in AAA management, while its full clinical impact will depend on addressing methodological, technical, and ethical challenges. Continued collaboration between clinicians, data scientists, and regulatory bodies will be essential to realize the vision of precision vascular care.

## 6. Conclusions

ML is transforming vascular surgery by enabling precise risk stratification, individualized treatment planning, and improved outcome prediction. In AAA management, particularly EVAR, ML models can integrate complex clinical and imaging data to predict survival, guide intervention strategies, and identify factors influencing aneurysm remodeling. Compared with traditional statistical methods, ML captures multidimensional interactions among variables such as nutritional status, immune function, comorbidities, and anatomical features, supporting nuanced patient stratification and personalized surveillance.

Challenges remain, including limited generalizability of single-center models, data quality issues, and lack of interpretability and external validation. Explainable AI techniques, such as SHAP and LIME, and multicenter datasets are critical for building clinician trust and ensuring robust performance.

Successful implementation will also depend on seamless integration into clinical workflows, harmonization of EHR data, compliance with regulatory standards, and effective interdisciplinary collaboration. As ML models become increasingly transparent, validated, and accessible, they are poised to play a central role in advancing precision vascular care.
